# Novel Effects of Statins on Cancer via Autophagy

**DOI:** 10.3390/ph15060648

**Published:** 2022-05-24

**Authors:** Daniela Mengual, Luz Elena Medrano, Wendy Villamizar-Villamizar, Estefanie Osorio-Llanes, Evelyn Mendoza-Torres, Samir Bolívar

**Affiliations:** 1 Healthcare Pharmacy and Pharmacology Research Group, Faculty of Chemistry and Pharmacy, Universidad del Atlántico, Barranquilla 081008, Colombia; dmengual@mail.uniatlantico.edu.co (D.M.); lemedrano@mail.uniatlantico.edu.co (L.E.M.); 2 Faculty of Health Sciences, Universidad Libre de Colombia, Barranquilla 081001, Colombia; wendyp-villamizarv@unilibre.edu.co; 3 Faculty of Natural and Exact Sciences, Universidad Libre de Colombia, Barranquilla 081001, Colombia; estefanie-osoriol@unilibre.edu.co

**Keywords:** statins, autophagy, cancer, signaling, carcinogenesis

## Abstract

Cancer is one of the main causes of death globally. Most of the molecular mechanisms underlying cancer are marked by complex aberrations that activate the critical cell-signaling pathways that play a pivotal role in cell metabolism, tumor development, cytoskeletal reorganization, and metastasis. The phosphatidylinositol 3-kinase/protein kinase-B/mammalian target of the rapamycin (PI3K/AKT/mTOR) pathway is one of the main signaling pathways involved in carcinogenesis and metastasis. Autophagy, a cellular pathway that delivers cytoplasmic components to lysosomes for degradation, plays a dual role in cancer, as either a tumor promoter or a tumor suppressor, depending on the stage of the carcinogenesis. Statins are the group of drugs of choice to lower the level of low-density lipoprotein (LDL) cholesterol in the blood. Experimental and clinical data suggest the potential of statins in the treatment of cancer. In vitro and in vivo studies have demonstrated the molecular mechanisms through which statins inhibit the proliferation and metastasis of cancer cells in different types of cancer. The anticancer properties of statins have been shown to result in the suppression of tumor growth, the induction of apoptosis, and autophagy. This literature review shows the dual role of the autophagic process in cancer and the latest scientific evidence related to the inducing effect exerted by statins on autophagy, which could explain their anticancer potential.

## 1. Introduction

Cancer is one of the most feared diseases of the 20th century and has shown a growing incidence in the 21st century [1]; it is also one of the leading causes of death worldwide. A GLOBOCAN study indicated that in 2020, there were 19.3 million new cases of cancer and almost 10 million deaths from cancer. While breast cancer surpassed lung cancer as the most commonly diagnosed type of cancer, with an estimated 2.3 million new cases (11.7%), lung cancer remained the leading cause of cancer death, with an estimated 1.8 million deaths (18%) [2].

Cancer denotes the uncontrolled proliferation of cells that possess metastatic properties [3]. These cells are characterized by changes in their activity, such as the suppression of apoptotic mechanisms, the disruption of adhesion, and the changes that occur as a result of genetic mutations [3]. The most common risk factors responsible for the appearance of cancer include tobacco and alcohol consumption, unhealthy diets, physical inactivity, and even air pollution [4]. Despite all the advances in the diagnosis of cancer and the therapeutic options available, treatment resistance remains a challenge for many cancer patients [5].

Many of the molecular mechanisms underlying cancer are marked by complex aberrations that activate critical cell-signaling pathways in carcinogenesis [6]. Cell-signaling pathways are critical for regulating cell metabolism, proliferation, tumor development and growth, cytoskeletal reorganization, and metastasis. One of the main signaling pathways involved in these processes is the phosphatidylinositol 3-kinase/protein kinase-B/ mammalian target of the rapamycin (PI3K/AKT/mTOR) pathway, which is closely related to the mitogen-activated protein kinase (MAPK), which is crucial and intensively studied in tumorigenesis [6,7,8,9].

Recent studies have found evidence that autophagy, a cellular pathway that delivers cytoplasmic components to lysosomes for degradation and recycling, contributes to treatment resistance in different types of cancer [10,11]. The role of autophagy in resistance to chemotherapies and targeted therapies has been described as being based largely on associations with various signaling pathways, including MAPK and PI3K/AKT signaling [5]. The regulation of autophagy by signaling pathways overlaps with the control of cell growth, proliferation, survival, and death [12]. Autophagy is activated in response to multiple stresses during cancer progression, such as nutrient starvation, endoplasmic reticulum (ER) stress protein response, and hypoxia; in addition, it is observed in the treatment of cancers with a broad spectrum of cytotoxic, chemotherapeutic, and targeted agents [13].

In cancer, autophagy plays a dual role through tumor promotion and its suppression [14]. In early tumorigenesis, autophagy prevents tumor initiation and suppresses cancer progression by removing oncogenic protein substrates, toxic unfolded proteins, and damaged organelles. However, in late tumorigenesis, autophagy, as a dynamic degradation and recycling system, contributes to the survival and growth of established tumors, increasing the aggressiveness of cancers by facilitating metastasis. This indicates that the regulation of autophagy can be used as an effective intervention strategy for cancer therapy [15]. 

In line with the vital role of autophagy in pathological conditions such as cancer, several drugs with modulating effects on this cellular mechanism are of therapeutic interest. Statins are a group of 3-hydroxy-3-methyl-glutaryl-coenzyme A (HMG-CoA) reductase inhibitors and are used to treat lipid disorders, since they inhibit the biosynthesis of cholesterol and isoprenoid metabolites, such as geranylgeranyl pyrophosphate (GGPP) and farnesyl pyrophosphate (FPP). Both GGPP and FPP participate in the post-translational events (isoprenylation) of various cell-signaling proteins, such as small members of the GTPase family [16]. Statins have pleiotropic properties, such as their antioxidant, anti-inflammatory, and antiproliferative effects, with applications in a multitude of pathologies [3]. In cancer patients, the efficacy of statins as anticancer agents has been evaluated both in monotherapy and in combination therapy with currently used chemotherapeutics [17]. Numerous in vitro and in vivo studies have demonstrated the molecular mechanisms through which statins inhibit the proliferation and metastasis of cancer cells and induce apoptosis in different types of cancer, such as breast [18], ovarian [19], lung [20], pancreatic [21], and hepatic [22] cancer, as well as gliomas [23].

The anticancer properties of statins have been shown to result from the suppression of tumor growth, the induction of apoptosis, and autophagy [19,24,25,26,27]. Statins can induce autophagy through the AMPK-mTOR signaling pathway, mainly through the cellular depletion of geranylgeranyl pyrophosphate (GGPP), which triggers autophagic responses [16]. The statin-induced activation of autophagy may play an important and potent role in reducing the anticancer effect of statins. In this review, we show the latest findings related to the role of statins as autophagy inducers and their effects on cancer. First, the dual role of the autophagic process in cancer is presented, followed by a description of the molecular bases involved in the inducing effect exerted by statins on autophagy, which could explain their anticancer properties. The relevant in vitro and in vivo experiments and clinical trials are also presented. 

## 2. Cancer

According to the World Health Organization (WHO), cancer is a generic term for a large group of diseases that can affect any part of the body [4]. A characteristic of cancer is the rapid proliferation of abnormal cells that can then invade other parts of the body and spread to other organs; this last process is called metastasis [4]. Metastatic disease is responsible for approximately 90% of cancer deaths [28]. Cancer can be detected through a laboratory test or a routine radiological test. In general, cancer must reach a size of 1 cm, or be composed of 1 million cells, before being detected; exceptions to this general rule include cancers of the blood and bone marrow, which often do not produce a mass but are detected through laboratory tests [1].

Cancer is a major cause of morbidity and mortality worldwide. Breast cancer has overtaken lung cancer as the most commonly diagnosed cancer, with an estimated 2.3 million new cases (11.7%), followed by lung (11.4%), colorectal (10.0%), prostate (7.3%), and stomach (5.6%) cancers. However, lung cancer remains the leading cause of cancer death, with an estimated 1.8 million deaths (18%), followed by colorectal (9.4%), liver (8.3%), stomach (7.7%), and breast (6.9%) cancers [2]. By 2040, the global burden of cancer is expected to be 28.4 million, an increase of 47% from 2020, and the number of cancer-related deaths is expected to rise to 16.4 million [29]. 

Cancer is caused by alterations in cell function due to the accumulation of many genetic and epigenetic changes within cells expressed in the accumulation of chromosomal or molecular aberrations, leading to genetic instability [30]. These genetic changes can occur due to errors in the timing of cell division, environmental factors, such as the chemicals in tobacco smoke and ultraviolet rays from the sun, diet, and excessive alcohol consumption [30]. There are more than 100 types of cancer; among the most common types are carcinomas, which are malignant epithelial tumors [31]. Sarcomas are a diverse group of cancers of mesenchymal origin, developing in bones and soft tissues, including muscles, fat, blood vessels, lymphatic vessels, and fibrous tissues [32]. Leukemias are malignant disorders of the blood and bone marrow that do not form solid tumors; instead, a large number of abnormal white blood cells accumulate in the blood and bone marrow, crowding out normal blood cells [33]. On the other hand, lymphoma is a type of cancer that begins in the cells of the lymphatic system; in total, 90% of lymphomas are of B cell origin, but they can also be T cells or natural killer cells; these can be broadly divided into non-Hodgkin and Hodgkin types [34].

Changes in metabolism are also among the main characteristics of cancer. Aerobic glycolysis and the activation of cell-signaling pathways play an important role in tumorigenesis, metastasis, drug resistance, and cancer stem-cell formation [35]. Although signaling pathways primarily regulate cell metabolism to maintain homeostasis, signaling in normal and oncogenic cells differs significantly, as it is well known that the deregulation of a pathway can lead to cancer progression and drug resistance [36]. Recent studies demonstrate that oncogenic signaling pathways such as PI3K/AKT/mTOR, the epidermal growth factor receptor (EGFR), Ras/MAPK, Notch, and Wnt/β-catenin, play a fundamental role in cancer therapy [37,38,39,40,41]. In addition to the oncogenic signaling pathway, there are also other tumor suppressor signaling pathways, which are repressed in cancer development and resistance to anticancer drugs [42,43,44,45,46].

The elucidation of the signaling pathways mentioned above gives a novel clue and an important contribution for the scientific community, yet there still is a lack of important advances in the development of effective cancer therapies [47].

While surgery and radiotherapy are the main treatments against local and non-metastatic cancers, the use of anticancer drugs (chemotherapy, as well as hormonal and biological therapies) is the new strategy to combat metastatic cancers. [47]. Chemotherapy induces damage in normal and rapidly dividing cells, including cells in the bone marrow, cells in the lining of the gastrointestinal and reproductive tracts, and hair-follicle cells [48]. Hormone therapy can cause a wide spectrum of complications, ranging from mild or moderate complications, such as thrombosis, hepatic steatosis, endometrial hypertrophy and osteoporosis, to severe complications, such as pulmonary embolism, avascular necrosis, intestinal perforation, and endometrial cancer [49].

A key challenge in the broad implementation of cancer immunotherapies is control over the modulation of the immune system, on which immunotherapies have serious adverse effects, including autoimmunity and nonspecific inflammation [50]. Significant advances have been made in cancer diagnosis and treatment in the last 50 years; previously, cancer was a death sentence, whereas there are currently drugs capable of curing various types of cancer. However, it is necessary to detect cancer at an early stage [1]. It is becoming increasingly clear that understanding the molecular profile of human tumors is essential for the effective use of anticancer drugs. The indiscriminate destruction of normal cells, the toxicity of conventional chemotherapeutic drugs, as well as the development of multiresistance, support the need to find new and effective targeted treatments based on changes in the molecular biology of tumor cells [47]. 

## 3. Autophagy 

In 2016, the Nobel Prize in Medicine was awarded to Japanese scientist Yoshinori Ohsumi for his research on autophagy and its impact on the study of human health and disease [51]. Autophagy is a cellular pathway that involves the degradation of multiple cytoplasmic components during standard physiological conditions and in response to different types of stress, including starvation [52]. In this pathway, cytosolic cargoes are sequestered in double-membrane vesicles called autophagosomes, which are delivered to lysosomes for degradation [53]. Autophagy is a fundamental mechanism in the maintenance of cellular homeostasis and is closely related to the appearance of a variety of human diseases [54]. 

**Figure 1:**
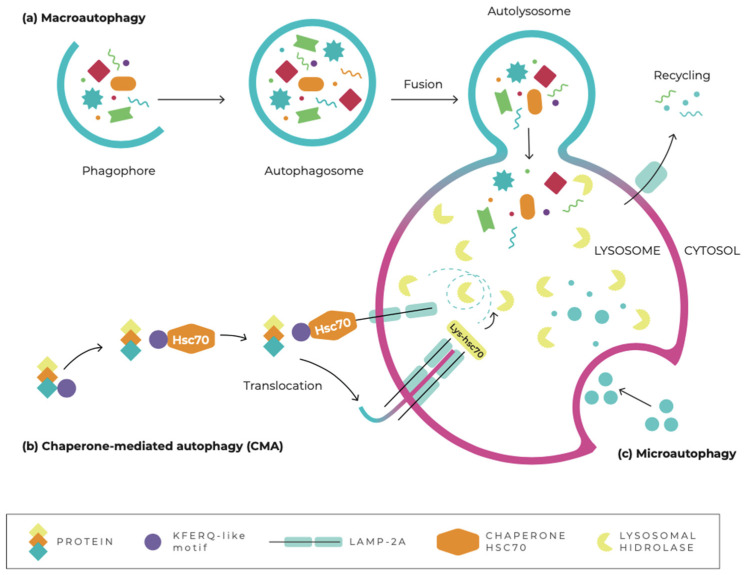
Schematic representation of the three subtypes of autophagy: (**a**) In macroautophagy, cytosolic cargo is sequestered by the expanding phagophore, leading to the formation of the autophagosome. Subsequently, the autophagosome fuses with the lysosomal membrane, forming the autolysosome, and releases the cytosolic cargo into the lysosome. (**b**) Chaperone-mediated autophagy is a process through which chaperone proteins such as Hsc70 recognize cytosolic cargo with a KFERQ-like motif. The chaperone–cargo complex associates with lysosome-associated membrane protein type 2A (LAMP-2A), resulting in translocation of the unfolded cytosolic protein into the lysosome. (**c**) Microautophagy refers to a process through which cytosolic cargo enters the lysosome through invagination or deformation of the lysosomal membrane without prior formation of an autophagosome. All three subtypes of autophagy lead to cargo degradation by lysosomal hydrolases, with the breakdown products ultimately released into the cytosol for reuse by the cell.

Autophagy can be divided into three main subtypes, according to the different mechanisms of removal of intracellular components: macroautophagy, microautophagy, and chaperone-mediated autophagy [55]. They promote the proteolytic degradation of the cytosolic components in the lysosome (Figure 1). Macroautophagy (hereafter referred to as autophagy), refers to the sequestration of cytosolic material, including proteins, lipids, and organelles, into double-membrane vesicles called autophagosomes, which subsequently fuse with lysosomes [53]. In chaperone-mediated autophagy (CMA), proteins containing a specific recognition sequence, KFERQ, delivered to lysosomes by molecular chaperones, such as the 70 kDa heat-shock analog protein (hsc70), are selectively degraded [56]. Microautophagy involves the delivery of autophagy substrates directly to lysosomes through the invagination of the lysosomal membrane [57]. 

Autophagy proceeds through a series of sequential steps, including autophagosomal initiation and nucleation (phagophore formation), the elongation of the phagophore, the maturation of the double-membrane autophagosome with charge assimilation, and, finally the fusion of the autophagosome to the lysosome (autolysosome formation), culminating in the degradation of the components within the autolysosomes and their release into the cytosol [58]. Each of these steps is regulated by a variety of molecular components, such as the following: the Unc-51-like autophagy-activating protein kinase complex (ULK1/2); the class III phosphatidylinositol 3-kinase (PI3KC3) protein complexes; phosphatidylinositol 3-phosphate (PI(3)P)-binding proteins and ATG9-containing membranes; the ATG12 and ATG8 ubiquitin-like conjugation systems (UBL), essential for the formation of autophagosomes; selective autophagy receptors and factors involved in autophagosome–lysosome fusion, such as SNARE proteins, small GTPases, anchoring factors, and others [59]. 

**Figure 2:**
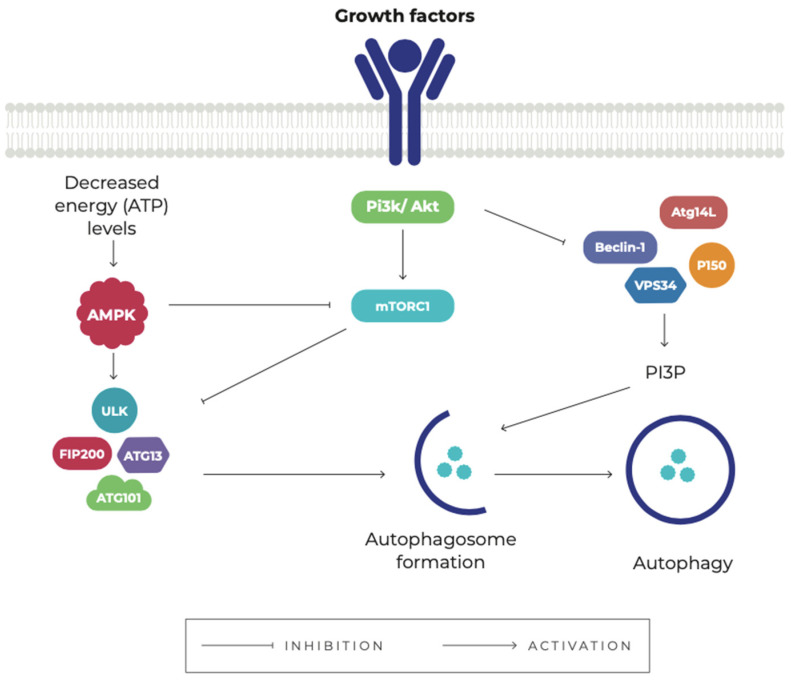
The main signaling pathways involved in the regulation of autophagy. (1) Phosphatidylinositol 3-kinase (PI3K)/Akt signaling activates mTORC1 in response to growth factors. The mTORC1 inhibits the Unc-51-like autophagy-activating kinase 1 complex (ULK1), thus repressing the autophagic mechanism. Akt can also negatively regulate autophagy through phosphorylation of the Beclin 1 complex. (2) Decreased energy (ATP) levels stimulate autophagy by activating AMPK, which negatively regulates mTORC1 and also phosphorylates the ULK1 complex. Autophagy is also regulated by the Beclin 1 interactive complex, which consists of Beclin 1, class III phosphatidylinositol-3-kinase (VPS34 or PI3KC3), P150, and ATG14L. This complex, when stimulated, generates phosphatidylinositol-3-phosphate (PI3P), necessary for the recruitment of autophagy-related proteins (Atg) associated with autophagosome formation.

Autophagy induction is tightly controlled by complex regulatory mechanisms that are induced by various stimuli, including hypoxia, nutrient starvation, infection by pathogens, growth factors, hormones, and oxidative stress [60]. Autophagy induced by nutrient and/or energy deprivation is mainly regulated by two signaling pathways, including the mammalian target of rapamycin (mTOR), and AMP-activated protein kinase (AMPK) [61] (Figure 2). 

mTOR is a highly conserved serine/threonine kinase that exists in two complexes, mTORC1 and mTORC2, each one with a specific function and location [62]. Through various mechanisms, mTORC1 signaling inhibits autophagy while promoting cell growth by stimulating biosynthetic pathways, including protein, lipid, and nucleotide synthesis [63]. AMPK is a key energy sensor and regulates a variety of metabolic processes including autophagy [64].

The PI3K/Akt/mTOR signaling pathway positively regulates mTOR [65] and the LKB1/AMPK/mTOR pathway acts mainly as a negative regulator of mTOR [66]. Evidence shows that mTOR plays a complex role in the induction, process, and termination of autophagy [67,68,69]; therefore, the inhibition of this protein is essential for the activation of autophagy. Several pharmacological interventions are available that inhibit mTOR (mTOR inhibitors), including rapamycin and its analogs [70]. Several drugs can also regulate autophagy by directly or indirectly activating AMPK, including metformin [71]. 

Recent evidence indicates that autophagy malfunction has been linked to a variety of human pathologies, including neurodegenerative diseases [72], cancer [15,73], cardiovascular diseases [74,75], respiratory conditions [76], liver diseases [77], infection, immunity and inflammation [78], kidney diseases [61,79], aging [80], and metabolic diseases, such as diabetes [81]. In addition, the accumulation of abnormal protein aggregates and dysfunctional mitochondria appears to be the basis of several neurodegenerative diseases, such as Alzheimer’s disease, Parkinson’s disease, and tauopathies [82,83]. Therefore, targeting the autophagy pathway and its regulatory components may eventually lead to the development of new treatments against the various diseases in which autophagy malfunctions. 

## 4. The Role of Autophagy in Cancer

In the last decade, numerous research groups have identified autophagy as a possible therapeutic target in various diseases, including cancer [84,85,86,87]. Autophagy can limit tumor cell growth or reduce mutagenesis or other damage caused by reactive oxygen species (ROS) by removing damaged mitochondria and other organelles [88]. However, it is well established that autophagy plays tumor-promoting and -suppressing roles. Its functions appear to depend on the tissue type, tumor stage, and genetic context, along with the activation of oncogenes and the inactivation of tumor suppressors [89]. In the early stages tumorigenesis, autophagy can prevent tumor initiation, proliferation, invasion, and metastasis; once tumors progress to a late stage, autophagy may contribute to tumor growth and survival, promote tumorigenesis, and cause resistance to therapeutic agents [15]. In this section, the different mechanisms involved in the regulation of autophagy during the carcinogenic process and metastasis are summarized, and therapeutic agents against cancers used as inhibitors or inducers of autophagy are discussed. 

### 4.1. The Role of Autophagy in Tumor Suppression

Several studies reveal that autophagy acts as a tumor suppressor [90,91,92]. In the early stages of cancer development, dysfunctional autophagy can cause the deficient degradation of damaged organelles and misfolded proteins; similarly, a deficiency in this pathway generates a greater production of ROS, which damages the DNA and creates genomic instability, in addition to the creation of a microenvironment of death and inflammation by necrotic tumor cells, which allows the development of carcinogenic processes [93]. The tumor-suppressive role of autophagy has been established by the finding that several human cancers harbor allelic deletions in pro-autophagy genes, such as Beclin-1, PTEN, AMPK, LKB1, and TSC1/2. On the other hand, the aberrant activation or expression of the oncogenes that activate mTOR, including PI3K, Akt, Ras, Rheb, and Bcl-2, results in the inhibition of autophagy [94]. The direct link between defective autophagy and the suppression of tumor development has been underlined by in vivo studies with genetically modified mice [95]. 

The Beclin-1 autophagy gene, a haploinsufficient tumor-suppressor protein, which is monoallelially deleted in breast, ovarian, and prostate cancers [96,97], and the Beclin-1 complex-binding proteins, such as the gene-associated protein resistance to UV radiation (UVRAG) and interaction factor with Bax-1 (Bif-1), may also act as tumor suppressors [98]. UVRAG promotes autophagy and suppresses the proliferation and tumorigenicity of cervical cancer cells and HCT116 human-colon cells [99]. Bif-1 has also been shown to regulate autophagy by forming a multiprotein complex with Beclin 1 via UVRAG; the loss of Bif-1 suppresses autophagic cell death and promotes tumorigenesis [100]. Another gene related to autophagy that is involved in cancer is p62; increased levels of this protein have been detected in several types of cancer, including breast cancer, prostate cancer, and hepatocellular carcinoma, among others [15], which highlights that the role of autophagy is crucial for tumor suppression by limiting the accumulation of p62.

Furthermore, it has been described that the alterations in genes related to autophagy (ATG) are associated with various types of cancer [94]. Evidence reveals that the monoallelic deletion of ATG7 and ATG5 leads to the development of multiple liver tumors from hepatocytes deficient in autophagy, due to damaged mitochondria and increased oxidative stress [101]. Other studies have identified several somatic mutations in ATG2B, ATG5, and ATG9B in gastrointestinal cancer [102,103]. Since these results suggest that alterations in autophagy promote the development of tumors in early stages, this mechanism of tumor suppression may suggest an important approach to cancer prevention. 

### 4.2. The Role of Autoaphagy as a Tumor Promoter 

It has been shown that autophagy favors the development, adaptation, and progression of tumor cells in advanced cancers [104,105,106,107]. Cancer cells show a greater dependence on autophagy than normal cells, although this depends on the context in which autophagy occurs and is probably due to the high metabolic and biosynthetic demands of dysregulated cancer cells [108]. The mechanism of autophagy-mediated tumor promotion is mainly based on eliminating ROS, damaged organelles, inhibiting DNA damage, maintaining genome stability, limiting inflammation, and preventing cancer cell damage under stress conditions [15]. Knocking down the expression of essential autophagy genes or deleting them in cancer cell lines can reduce the development of tumorigenesis, which demonstrates the functional importance of autophagy in tumor promotion [109]. 

The functional state of autophagy is clearly influenced by the tumor microenvironment and the immune system, which reveals the importance of evaluating autophagy in genetically modified mouse cancer models (GEMM) [91]. The inhibition of autophagy through the deletion of ATG5 decreased the ability of premalignant lesions to develop into invasive cancer in mice with KRAS-driven pancreatic cancer with loss-of-heterozygosity (LOH) for Trp53 (tumor suppressor protein) [109]. In a KRas-dependent model of spontaneous non-small-cell lung cancer (NSCLC), the inhibition of autophagy through ATG7 decreased tumor growth, converting adenomas and carcinomas into a benign disease characterized by the accumulation of defective mitochondria [110,111]. Tumors may be more dependent on autophagy than most normal tissues; therefore, the inhibition of autophagy may be an important therapeutic strategy for cancer therapy and prevention [91]. 

### 4.3. Autophagy as a Regulator of Cancer Metastasis 

Metastasis is the cause of 90% of cancer deaths in humans [28]. Tumor cells grow in tight spaces and separate from the primary tumor under stress conditions, after which the migrating cancer cells become metastatic nodules [112]. Numerous cellular processes are essential for cancer metastasis, including extracellular matrix (ECM) degradation, epithelial-to-mesenchymal transition (EMT), tumor angiogenesis, the development of an inflammatory tumor microenvironment, and cell dysfunction due to programmed cell death machinery [113]. Autophagy plays a complex role in cancer metastasis, since scientific evidence has shown the prometastatic and antimetastatic functions of autophagy [114]. During the initial stage of metastasis, autophagy can act as a suppressor of this process by restricting tumor necrosis and inflammation, as well as reducing the invasion and spread of cancer cells from primary sites [113]. However, during the advanced stages, autophagy can act as a promoter of metastasis, promoting cancer cell survival and colonization at distant sites [114]. 

In the early stages, autophagy can reduce metastatic progression from the primary tumor site, mainly by restricting the inflammatory response [114]. Autophagy induces the activation of the anticancer immune response mediated by dendritic cells, through the release of high-mobility group box protein 1 (HMGB1) in cancer cells [115]. On the other hand, a study showed that the blockage of the CXCR4/mTOR signaling pathway induces autophagic cell death and exhibits antimetastatic properties in gastric cancer cells [116]. In 2020, Marsh et al. demonstrated that the accumulation of the neighboring protein of gene 1 BRCA1 (NBR1) in autophagy-deficient cells causes a greater propensity for spontaneous metastasis and, in addition, the inhibition of genetic autophagy in multiple models of autophagy, while breast cancer accelerates the proliferation of disseminated tumor cells (DTC) in macro-metastases [117,118]; the pharmacological and genetic induction of autophagy suppresses pro-metastatic differentiation and metastatic growth. 

### 4.4. Autophagy as a Target for Cancer Therapy

Autophagy provides a mechanism that allows cancer cells shed from the extracellular matrix (ECM) to acquire resistance to anoikis to survive and travel through the circulatory and lymphatic systems to spread throughout the body [119]. Several in vitro studies associate autophagy with supporting the pro-metastatic mechanism of tumor cells, such as adhesion-independent survival, metabolic adaptation, cell invasion and motility [120,121,122]. It has been established that autophagy is critical for cancer stem cell (CSC) maintenance and drug resistance and with sustaining the dynamic balance between CSC and non-CSC [113]. Similar studies indicate that the inhibition of autophagy reduces the metastasis of hepatocellular carcinoma (HCC), reducing resistance to anoikis and lung metastasis of HCC cells [123]. Other studies have identified an association between increased autophagy and metastasis in various types of cancer, including metastasis from breast cancer [124], prostate cancer [125] and colorectal cancer [126]. These evidences indicate that autophagy plays an essential role in the metastatic transformation of tumors and increases the aggressiveness of cancer cells.

The modulation of autophagy plays a dual role in tumor induction and suppression in many types of cancer [127]. Targeting autophagy may enhance the beneficial effects of many cancer therapies [108]. Several regulators of autophagy, such as rapamycin and its analogs (temsirolimus and everolimus), 3-methyladenine (3-MA), chloroquine (CQ), hydroxychloroquine (HCQ), and spautin-1, are used in the treatment of cancer [128,129,130,131]. Rapamycin (RAPA, sirolimus) is a naturally occurring mTOR inhibitor and its analogs (rapalogs), everolimus and temsirolimus, inhibit mTORC1 and induce autophagy, and, consequently, may contribute to a reduction in tumor growth [132]. Rapamycin has been shown to effectively inhibit HEC-1A and Ishikawa human endometrial cancer cell proliferation through the downregulation of AKT/mTOR phosphorylation [133]; similarly, there are studies confirming the efficacy of everolimus in the treatment of advanced pancreatic neuroendocrine tumors [134] and metastatic (HR+)/HER2-negative breast cancer [135,136]. Temsirolimus is currently used to treat advanced or metastatic renal cell carcinoma [137]. 

Current clinical efforts in cancer therapy are focused on the use of FDA-approved chloroquine (CQ) or hydroxychloroquine (HCQ) usage [138]. Both HCQ and CQ have been used as standard autophagy inhibitors in many preclinical and clinical studies. In clinical trials, HCQ has been used as an autophagy inhibitor for the treatment of advanced cancers [139,140], while preclinical studies have shown that CQ or HCQ can reduce tumor cell growth by inhibiting autophagy in cancer, bladder cancer, pancreatic cancer, lung cancer, and glioma [141,142,143,144]. Similarly, several studies report that CQ can improve the efficacy of chemotherapy and radiotherapy by inhibiting autophagy [145,146]

On the other hand, 3-methyladenine (3-MA) is one of the most widely used autophagy inhibitors. This compound affects two of the molecular targets involved in the autophagy process: phosphatidylinositol 3-kinase (PI3K) and Vps34 [132]. In many types of cancer, 3-MA improves the efficacy of chemotherapy or radiation. For example, the inhibition of autophagy using 3-MA increased the cytotoxicity of radiotherapy on human esophageal squamous cell carcinoma [147]. Likewise, in another study, it was shown that the inhibition of autophagy by 3-MA promotes hypoxia-induced apoptosis in human colorectal cancer cells [130]. 

The potent and specific autophagy inhibitor-1 (Spautin-1) is a molecule that inhibits autophagy by inducing the degradation of beclin-1, a protein required for the initiation of autophagy [148]. Spautin-1 is used for the treatment of prostate cancer by inhibiting EGFR signaling [149]. Other studies have shown that Spautin-1 ameliorated the pathogenesis of pancreatitis through the inhibition of autophagy [150]. In addition, it was shown that Spautin-1 inhibits imatinib-mesylate-induced autophagy in chronic myeloid leukemia cells by downregulating Beclin 1 [151]. It was shown that Spautin-1 promoted cell apoptosis by activating glycogen synthase kinase 3 beta (GSK3β) via PI3K/AKT, which reduced the antiapoptotic protein Mcl-1 [151]. These data indicate a possible therapeutic application of the inhibitory effect of Spautin-1 on cancer cells.

Autophagy has emerged as a promising therapeutic strategy for the treatment of tumors. Thus, the induction of autophagy may be favorable for cancer chemoprevention in normal cells. Furthermore, the inhibition of the autophagic pathway can be combined with cytotoxic drugs to achieve higher efficacy, representing a valuable therapeutic strategy to overcome resistance to anticancer drug therapies. Therefore, the dual role of autophagy as an inducer and inhibitor is important in determining cancer treatment, since treatment depends on the type of tumor, the stress signal, and other circumstances.

## 5. Statins

Statins are lipid-lowering drugs that prevent endogenous cholesterol synthesis due to the partial and reversible competitive inhibition of the enzyme hydroxymethyl glutaryl CoA reductase (HMG-CoA reductase), which participates in the mevalonate pathway, an important precursor in the synthesis of cholesterol; however, although this is their main mechanism of action, they have other mechanisms that contribute to the same purpose, such as: increasing LDL receptors, decreasing the release of lipoproteins from the liver to the periphery, increasing the elimination of LDL, reducing the production of lipoproteins, and reducing triglyceride levels by increasing the elimination of VLDL. The pharmacological effects of these mechanisms include reductions in total cholesterol by 20–40% and LDL cholesterol (bad cholesterol) by 20–60%, increases in HDL cholesterol (good cholesterol) of 2–16% and, additionally, decreases in triglycerides of 10–40%. These ranges depend on the active ingredient used, the patient, and the dose prescribed [152,153,154,155,156,157]. 

It is necessary to highlight that statins have some effects that favor vascular health, such as their anti-inflammatory properties, their reduction of cardiovascular morbidity and mortality in hyperlipidemia, and their ability to induce regression in coronary atherosclerosis and decrease the risk of cerebrovascular accidents [152,154,155]. Statins can be used as preventive or therapeutic measures in all types of hyperlipidemias in which LDL values are increased, such as in hypercholesterolemia and in mixed dyslipidemia; they are very useful for patients with vascular disease and for patients with very high LDL, as well as in moderate cases. In terms of their pharmacokinetics, statins generally require minimal kidney elimination, so they can be used in patients with kidney failure [152,157]. 

Statins can be classified into three groups, according to their efficacy, which depends on the dosage. Some examples of this are rosuvastatin, which can be highly effective (20–40 mg) or moderate (5–10 mg), and atorvastatin (high: 40–80 mg; moderate: 10–20 mg). Other statins that are classified as being moderate and low in efficacy, according to the dose, are: simvastatin, lovastatin, pravastatin, fluvastatin, and pitavastatin [152]. 

The side effects of statins that occur most frequently are musculoskeletal symptoms, such as myalgia (5–15%), that are usually symmetrical and located in the legs, where they can occur together with elevated creatine kinase (CK), and gastrointestinal, including abdominal pain, constipation, flatulence, nausea, diarrhea, and headache and fatigue. Another, less frequent, side effect is liver toxicity (2%), which is evidenced by a generally mild increase in hepatic aminotransferases and, therefore, does not require the discontinuation of statins and is dose-dependent; to control this side effect, liver enzyme tests are recommended before starting statin therapy and when it is clinically relevant during treatment, such as to treat the symptoms of hepatitis [152,153,154]. The most potentially serious adverse effect is rhabdomyolysis (infrequent), which generates myoglobinuria and renal failure, which is more frequent in elderly people with generalized weakness, patients with previous renal insufficiency, shock, patients with concomitant disorders, patients who are polymedicated, patients with hypothyroidism, and in perioperative periods [152,156]. Typically, it is not necessary to perform routine CK, but it is necessary to request this test in the presence of muscle pain, while the medication is suspended [154]. 

Some interactions that can cause side effects of the musculoskeletal type by inhibiting the catabolism of statins, thereby increasing their plasma concentration, occur with drugs that are metabolized in the cytochrome P450 system, such as antifungals (ketoconazole, itraconazole), macrolides (clarithromycin and erythromycin), and other drugs that increase the risk of myopathies, such as fibrates (gemfibrozil), cyclosporine, digoxin (digitalis), calcium channel blockers (verapamil and diltiazem), colchicine, and amiodarone and protease inhibitors. Some factors that also increase the risk of myopathies are grapefruit juice intake, intense physical exercise, and high alcohol intake. The diagnosis of myalgias due to statins is clinical, based on the disappearance of these symptoms when the statin is discontinued and their reappearance when resuming its consumption [152,154].

Statins are contraindicated in patients with significant liver dysfunction, in pregnancy, and in ongoing lactation; they can also increase the risk of type 2 diabetes mellitus in patients who have a predisposition to this pathology, as is the case of subjects with metabolic syndrome and prediabetics. This situation has been described when high doses are consumed; therefore, non-pharmacological treatment should be encouraged (diet, exercise), and it is usually continued because the reduction in cardiovascular risk outweighs the possible effect of altering glycemic homeostasis [152,153,154].

Various preclinical studies to date have shown that statins have the ability to effectively induce cell death in different types of human tumor cell. Therefore, statin use is currently an active field of research, within which clinical trials are being developed to establish more clearly whether the use of statins in cancer patients would be beneficial [157]. 

### 5.1. Statins and Autophagy 

**Figure 3:**
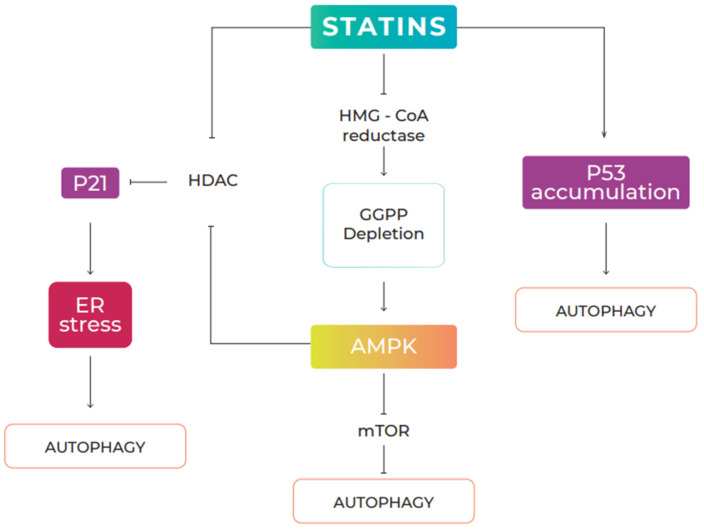
Statin-induced autophagy signaling pathways. Statins regulate autophagy in several ways: (1) By inhibiting HMG-CoA reductase, statins interfere with the production of mevalonic acid, a precursor in the biosynthesis of geranylgeranyl pyrophosphate (GGPP). Depletion of cellular levels of geranylgeranyl pyrophosphate induces AMPK signaling, repressing mTOR activity, leading to activation of autophagy; (2) statins induce p21 expression through inhibition of histone deacetylase (HDAC) activity, through direct interaction or AMPK-mediated phosphorylation. Activation of the AMPK/p21 signal induced by statins generates ER stress and induces autophagic responses. (3) Statins can increase the accumulation of nuclear p53 and induce autophagy in a p53-dependent manner.

Evidence has shown that statins can induce autophagy in various types of cell, including vascular endothelial, cardiac, and mesenchymal cells of the respiratory tract, as well as in transformed cells, such as tumor cells [26,158,159,160]. Statins are a group of drugs that have been used for a long time as cholesterol-lowering agents. They act by inhibiting 3-hydroxy-3-methyl-glutaryl-CoA reductase, a limiting enzyme of the mevalonate pathway. In this pathway, statins also inhibit a variety of other intermediate metabolites, including the formation of isoprenoids, such as farnesyl pyrophosphate (FPP) and geranylgeranyl pyrophosphate (GGPP), leading to the inhibition of the isoprenylation of small GTPases, such as Ras, Rac, Rab, and Rho [161]. Several signaling pathways have been implicated in the regulation of statin-mediated autophagy, including the AMPK/mTOR (AMP-activated protein kinase/mechanistic target of rapamycin) pathway and the AMPK/p21 pathway. Finally, it has been suggested that p53 accumulation induced by statins can activate autophagy [160,162,163] (Figure 3). 

Numerous molecular mediators are involved in autophagy, including the prenylation of GTPase proteins that are indicated as ATG proteins: Rabs, RalB, and Rheb [164]. Rabs are proteins that act as molecular switches, mediating the transport and fusion of vesicles; they are necessary to develop subdomains in membranes to facilitate maturation [165]. Recent evidence has shown that some Rabs, including Rab1, Rab5, Rab7, Rab8A, Rab8B, Rab9, Rab11, Rab23, Rab24, Rab25, Rab3, and Rab33B, are essential for autophagy [166,167,168]. The GTPase, RalB, controls crucial physiological processes, including autophagy and invasion. It localizes to a nascent autophagosome and is activated upon nutrient deprivation. Due to its binding to its effector, Exo84, RalB induces the assembly of ULK1 and Beclin1-VPS34, which are necessary for the formation and maturation of autophagosomes [169]. On the other hand, the brain-enriched Ras homologue (Rheb) regulates cell growth, proliferation, and regeneration through the activation of mTORC1, which results in the inhibition of autophagy by inhibiting the kinase activity of ULK; under starvation conditions, Rheb is inactivated by the Rheb activator protein, GTPase, and mTORC1 is inactivated, leading to the induction of autophagy [170]. Thus, Rheb inhibition causes statin-induced autophagy.

Another study demonstrated that atorvastatin induced autophagy by enhancing Beclin1 and LC3-II gene expression [171] and through the AMPK/mTOR pathway [16]. Similar results were evidenced in endothelial progenitor cells, in which pravastatin enhanced autophagy activity through the upregulation of LC3-II/Beclin-1 and autophagosome formation [158]; and another study also demonstrated that autophagic modulation by rosuvastatin prevents rotenone-induced neurotoxicity, because rosuvastatin treatment alone increased levels of mTOR-independent/upstream autophagy markers, including Beclin-1 and AMPK [172]. On the other hand, Wei et al. (2013) observed that simvastatin increased autophagy by inhibiting the Rac1-mTOR pathway [159]. In primary cultures of human airway smooth muscle (HASM) cells and human atrial fibroblast (hAF) cell lines, statin-induced autophagy and apoptosis were shown to be p53-dependent [160]. In addition, in vitro tests indicated that statins such as lovastatin and simvastatin caused the degradation of S-phase-associated protein kinase 2 (SKP2) and, consequently, an increase in Beclin1 levels and autophagy [173,174]. 

In two lung adenocarcinoma cell lines, fluvastatin stimulated autophagy activation by increasing LC3-II levels [175]. Similarly, Ghavami et al. (2012) showed that statins induce apoptosis and autophagy in the mesenchymal cells of the human lung, and also suggested that autophagy has a crucial role in determining the degree of stress of the endoplasmic reticulum (ER), unfolded protein response (UPR), and cell-line permissiveness (hAF) to statin-induced death [176]. Another study indicated that simvastatin induced autophagy in cardiac cell lines and in mouse hearts in vivo; these cells were treated with simvastatin, producing a slight mitochondrial depolarization relative to controls and a significant increase in PTEN levels. This shows that simvastatin is capable of inducing PTEN-mediated mitochondrial autophagy, which is related to its cardioprotective capacity [177].

Some studies have shown that statins can inhibit autophagy. For example, atorvastatin inhibits vascular endothelial cell autophagy, an effect that may be related to atorvastatin’s role in improving endothelial function. However, the use of atorvastatin, before the onset of induced autophagy, cannot effectively inhibit autophagy [178]. Another study showed that the inhibition of the mevalonate pathway simultaneously blocks autophagosome maturation, leading to reduced autophagic flux [179]. Similarly, one study reported that autophagic flux block is caused by reduced prenylation and, more specifically, the geranylgeranylation of Rab11, a small GTPase required for autophagosome formation, which acts in the recycling endosome [180]. Thus, mevalonate pathway activity functions as a metabolic requirement for basal autophagic flux through Rab11 geranylgeranylation.

Taken together, these studies demonstrate that the role of statins in autophagy is related to the expression levels of GGPP and prenylated proteins, making them important players in this mechanism. Therefore, the therapeutic effects of statins in various cell types have been attributed to the modulation of autophagy, which is essential for maintaining cellular homeostasis and explains the elimination of unfavorable cells or specific organelles within cells.

### 5.2. Effects of Statins on Cancer via Autophagy 

Statins have emerged as a potential therapy against cancer due to their effects on autophagy [181]. Several studies have shown that the role of autophagy in tumorigenesis is very contradictory, since its induction could accelerate tumor progression or, on the contrary, induce tumor suppression [175]. In accordance with the important role that autophagy plays in the pathological conditions of cancer, compounds with proautophagic or antiautophagic modulating effects are of pharmacological interest [181].

In preclinical studies, statins have been shown to be capable of inducing autophagy in some cancer cells by inhibiting geranylgeranyl [26,182,183]. The antitumor activity of atorvastatin (ATO) has been shown to be associated with the induction of autophagy in breast cancer cells [25,184] and ovarian cancer [19]. ATO induces autophagy in PC3 prostate cancer cells by activating LC3 transcription [185]. Sheng et al. (2020) showed that ATO inhibited tumor growth and promoted the apoptosis of cervical cancer both in vitro and in vivo, which could be associated with the suppression of the mevalonate pathway. Furthermore, ATO could also induce autophagy in cervical cancer cells, but, more importantly, the pharmacological inhibition of autophagy significantly enhanced ATO-induced cytotoxicity in cervical cancer [24]. Similarly, Yan et al. (2014) showed that ATO induces autophagy in Huh7 and HCT116 gastrointestinal tumor cell lines and that combinations of ATO with autophagy inhibitors provide a novel and promising strategy to improve the treatment of digestive malignancies [182]. Another study suggests that the inhibition of autophagy potentiates ATO-induced apoptotic cell death in human bladder cancer cells [186] (Table 1).

Fluvastatin has been reported to induce autophagy in breast cancer cells through AMPK phosphorylation and the inhibition of basal extracellular signal-regulated kinase (ERK) phosphorylation [187]. Fluvastatin can suppress the bone metastasis of lung adenocarcinoma by inducing nuclear wild-type p53 expression, activating AMPK-mTOR-dependent autophagy in cancer cells [175]. Fluvastatin induced apoptosis in lymphoma cells by regulating autophagy through the inhibition of metabolic products of the HMG-CoA reductase reaction [188] (Table 2). Asakura et al. (2011) studied the antitumor activity of lovastatin in malignant pleural mesothelioma cells. Their results demonstrated that lovastatin administration reduced primary tumors and metastasis in a NOG mouse model of human malignant mesothelioma. In vitro studies demonstrated that lovastatin administration induced cytostatic effects, such as reduced cell viability and cell migration, in ACC-MESO-1 cells. The authors suggested that these effects were dependent on autophagic changes rather than apoptosis; furthermore, the induction of autophagic changes by lovastatin in ACC-MESO-1 cells was independent of mTOR and was considered to be dependent, at least in part, on Rac/phospholipase C/inositol 1,4,5-triphosphate [189]. Wojtkowiak et al. (2011) found that the combination of lovastatin and a farnesyl transferase inhibitor (FTI-1) induced a dysfunctional autophagic program and non-apoptotic cell death in malignant liver sheath tumor cell lines in human peripheral nerves [190]. Another study showed that the combination of lovastatin and cisplatin improves LC3B-II levels and reduces the viability of cancer cells by inducing autophagic cell death [191]. On the other hand, a recent study indicated that lovastatin decreases the survival of PEL cells, an aggressive B-cell lymphoma, by phosphorylating ERK1/2, blocking autophagic flux [192] (Table 3).

Researchers have shown that pitavastatin, another class of statins, in conjunction with metformin could preserve mitochondrial function, activate AMPK, and inhibit PI3K/mTOR, unlike treatment with metformin or pitavastatin alone. These findings clearly indicated that metformin plus pitavastatin had a synergistic anticancer effect on pancreatic cancer cells, which was potentially caused by the activation of AMPK and the inhibition of PI3K/mTOR signaling [193]. The antitumor activity of pitavastatin was also investigated in melanoma cells; the results demonstrated that melanoma cells treated with combined pitavastatin–dacarbazine (DTIC) expressed a high level of LC3-II, a marker of autophagy. The chemical inhibition of autophagy resulted in enhanced cell viability, suggesting that pitavastatin–DTIC-induced autophagy occurs as a mechanism of cell death. 

In support of this, it has been suggested that DTIC and pitavastatin may have a role in the induction of autophagy, specifically as a mode of cell death. Therefore, the pitavastatin–DTIC combination treatment provides a synergistic anticancer effect through apoptosis and autophagy [194] (Table 4). On the other hand, a study showed that treatment with rosuvastatin can be an alternative for patients with papillary thyroid cancer. Rosuvastatin induced autophagic changes in papillary thyroid carcinoma (B-CPAP) cells, even at low doses, showing a variation from autophagic changes to apoptosis with increasing concentrations of rosuvastatin. In normal thyroid cells (Nthy-ori3-1), however, minimal/early autophagic changes were observed only at higher doses and with increased exposure times. The results indicated that rosuvastatin treatment induced autophagy and subsequent apoptosis in B-CPAP cells [195] (Table 5).

Castellanos et al. (2018) have shown that simvastatin (SIM) mainly induces cell apoptosis, while pentoxifylline (PTX) induces autophagy and cell apoptosis; the two drugs used in combination reduced autophagy rates while apoptosis levels increased inversely. These results strongly support the existence of a well-regulated balance between apoptosis and autophagy. Thus, the study most likely revealed an interesting pharmaceutical property of simvastatin, indicating that it is capable of disrupting equilibrium, altering cell fate, and inducing cell death by stimulating extracellular regulated protein kinases 1 and 2 (ERK1/2) and Akt pathways. The results therefore suggest that the induction of autophagy may be a protective mechanism preventing the death of MDA-MB-231 breast cancer cells and that the combined use of PTX and SIM may render quiescent autophagic cancer cells experiencing apoptosis. Therefore, this may be a new treatment strategy for triple-negative breast cancer [196].

The role of autophagy in glioma cell death induced by the drug simvastatin has been investigated, suggesting that the inhibition of the AMPK-dependent autophagic response could sensitize glioma cells to apoptotic death induced by simvastatin [197]. The anticancer effects of the chemotherapeutic agent temozolomide (TMZ), a drug used for the treatment of glioblastoma (GBM), is significantly reduced by autophagy induced by TMZ. One study demonstrated that simvastatin inhibits TMZ-induced autophagic flux by blocking autophagosome–lysosome formation, thus sensitizing glioblastoma cells to TMZ-induced cell death, making it a promising therapeutic strategy for GBM treatment [198] (Table 6).

In vitro and in vivo investigations indicate that a number of beneficial effects of statins are due to mechanisms that predominantly interfere with tumor growth and invasion. Autophagy, a self-degradation pathway, acts as a double-edged sword in cancer. Statins can induce autophagy in some cancer cells. Statins have been shown to inhibit the cell viability of multiple cancers, including ovarian cancer, lung adenocarcinoma, malignant pleural mesothelioma, melanoma, and pancreatic cancer by inducing autophagy. The combination of statins with autophagic inhibitors is a new and promising strategy to improve the treatment of digestive malignancies, cervical cancer, and bladder cancer. This means that autophagy plays a fundamental role in pathological conditions. However, more studies are needed in the future to fully understand the modulatory effect of statins on autophagy.

## 6. Clinical Trials 

In light of the positive results of in vivo and in vitro studies using statins as potential treatments for cancer, the performance of clinical trials has been increasing. Clinical trials are always sources of new evidence as they provide detailed information on how groups of drugs are tested and given to selected populations with different clinical conditions. Table 7, Table 8 and Table 9 summarize the results of clinical trials with different statins alone or combined with other medications for the treatment of cancer. 

**Table 7:** Clinical trials (phase 2) with the use of atorvastatin for the treatment of cancer.

Trial Name (Identifier)	Patient Population (Condition)	Treatment Groups	Enrollment (Participants)	Sponsor
Donor Atorvastatin Treatment in Preventing Severe Acute GVHD After Nonmyeloablative Peripheral Blood Stem Cell Transplant in Patients With Hematological Malignancies (NCT01527045) [199]	Hematological malignancies	Drug: Atorvastatin calcium Drug: Cyclosporine Drug: Fludarabine phosphate Drug: Mycophenolate mofetil Procedures: Nonmyeloablative allogeneic hematopoietic stem-cell transplantation and peripheral blood stem-cell transplantation. Radiation: Total-body irradiation	47	Fred Hutchinson Cancer Research Center
Safety & Efficacy of Atorvastatin for Prophylaxis of Acute Graft Versus Host Disease in Patients With Hematological Malignancies HLA- Donor Hematopoietic Stem Cell Transplantation (NCT01491958) [200]	Acute myelogenous leukemia Acute lymphocytic leukemia Myelodysplastic syndrome	Drug: Atorvastatin Drug: Tacrolimus Drug: Methotrexate	40	Ohio State University Comprehensive Cancer Center
Pilot Study of Statin Therapy in Young Adult Survivors of Childhood Cancer [201] (NCT01733953)	Cardiovascular disease Childhood ALL Childhood NHL	Drug: Atorvastatin Drug: Sugar pill (placebo)	27	University of Minnesota
Atorvastatin Calcium and Celecoxib in Treating Patients With Rising PSA Levels After Local Therapy for Prostate Cancer (NCT01220973) [202]	Prostate cancer	Drug: Atorvastatin calcium Drug: Celecoxib Other: Laboratory Biomarker analysis	27	Rutgers, The State University of New Jersey

**Table 8:** Clinical trials including simvastatin for the treatment of cancer.

Trial Name (Identifier)	Phase	Patient Population (Condition)	Treatment Groups	Enrollment (Participants)	Sponsor
Study to Assess the Effect of AZD9291 on the Blood Levels of Simvastatin in Patients With EGFRm+ NSCLC (NCT02197234) [203]	1	Non-small-cell lung cancer	Procedure: Pharmacokinetic sampling—AZD9291. Drug: Simvastatin Drug: AZD9291 tablet dosing.	52	AstraZeneca
Simvastatin in Preventing a New Breast Cancer in Women at High Risk for a New Breast Cancer (NCT00334542) [204]	2	Breast cancer	Drug: Simvastatin	50	Sidney Kimmel Comprehensive Cancer Center at Johns Hopkins
Pre-Operative Statin Therapy Versus Placebo in Human Prostate Cancer (NCT00572468) [205]	N/A	Prostate cancer	Drug: 40 mg of Simvastatin Other: Placebo	42	VA Office of Research and Development
Detection and Prevention of Anthracycline-Related Cardiac Toxicity With Concurrent Simvastatin (NCT02096588) [206]	2	Breast cancer Stages I, II, and III of breast cancer	Drug: Simvastatin Drug: Doxorubicin/ cyclophosphamide	34	Avon Foundation

**Table 9:** Clinical trials including different statins for the treatment of cancer.

Trial Name (Identifier)	Phase	Patient Population (Condition)	Treatment Groups	Enrollment (Participants)	Sponsor
Study of Effectiveness of Lovastatin to Prevent Radiation-Induced Rectal Injury (NCT00580970) [207]	2	Prostate cancer	Drug: Lovastatin	73	Virginia Commonwealth University
Phase 2 Study of Lovastatin as Breast Cancer Chemoprevention (NCT00285857) [208]	2	Breast cancer	Drug: Lovastatin	30	Stanford University
Study to Assess the Effect of AZD9291 on the Blood Levels of Rosuvastatin, in Patients with EGFRm+ Non-small Cell Lung Cancer (NCT02317016) [209]	1	Non-small-cell lung cancer	Procedure: Pharmacokinetic sampling—AZD9291 Drug: AZD9291 tablet dosing Drug: Rosuvastatin	44	AstraZeneca
Rosuvastatin to Lower Circulating Tissue Factor Bearing Microparticles in Metastatic Breast Cancer (NCT01299038) [210]	2	Breast cancer	Drug: Rosuvastatin	20	Beth Israel Deaconess Medical Center
Idarubicin, Cytarabine and Pravastatin (IAP) for Induction of Newly Diagnosed Acute Myeloid Leukemia (NCT01831232) [211]	N/A	Acute myeloid leukemia	Drug: Pravastatin sodium Drug: Idarubicin Drug: Cytarabine Other: Laboratory biomarker analysis	24	Fred Hutchinson Cancer Research Center

In the study, “Safety & Efficacy of Atorvastatin for Prophylaxis of Acute Graft Versus Host Disease in Patients With Hematological Malignancies HLA-Donor Hematopoietic Stem Cell Transplantation”, identified by the code NCT01491958 [200], the results obtained were as follows: Out of thirty-six patients on whom the safety of atorvastatin for transplant recipients was tested in terms of adverse reactions and toxicities, two presented a grade 2 elevation in liver enzymes and one a grade 4; the time to neutrophil engraftment was 18 days and the time to platelet engraftment was 14 days; 30% of patients developed grade II to IV acute graft-versus-host disease on day +100 of atorvastatin administration; of the 34 patients analyzed, 43% developed chronic graft-versus-host disease up to 1 year after transplantation, and five of thirty-six patients presented serious adverse events when safety of atorvastatin was analyzed. 

In the “Simvastatin in Preventing a New Breast Cancer in Women at High Risk for a New Breast Cancer” study, identified by the code, NCT00334542 [204], no all-cause mortality or serious adverse events were recorded in the study participants. In total, 34% of the participants presented non-serious adverse events, including constipation, muscle weakness, myalgia, headache, maculopapular rash. 

In 2011, a retrospective observational study was carried out called “Combination Statin, Acetylsalicylic Acid and Dutasteride Use in Prostate Cancer”, identified by the code, NCT01428869 [212], in which 8231 participants were included. Its purpose was to evaluate whether there was any interaction between statins, acetyl salicylic acid, and dutasteride in the protection against prostate cancer, the development of high-grade prostate cancer, or symptoms of the lower urinary tract. So far, no results have been reported. 

## 7. Perspectives and Conclusions 

Statins act by inhibiting the enzyme HMG-CoA reductase, producing, as pharmacological effects, reductions in cholesterol levels and the prevention of cardiovascular diseases. In recent years, there has been a strong interest in the molecular mechanisms associated with satins’ “pleiotropic” effects beyond their reduction of lipoproteins. Consequently, different investigations have recently shown that statins can modulate several of the molecules involved in the autophagy pathways in tumor cells, as well as molecules in the vascular endothelial, cardiac, and mesenchymal cells of the respiratory tract.

Autophagy, as a self-adaptive strategy, performs functions of tumor promotion and suppression. These functions appear to depend on the tissue type, tumor stage, and genetic context, along with the activation of oncogenes and the inactivation of tumor suppressors. In the early stages of tumorigenesis, autophagy can prevent tumor initiation, proliferation, invasion, and metastasis; once tumors progress to a late stage, autophagy may contribute to tumor growth and survival, promote tumorigenesis, and cause resistance to therapeutic agents. Many studies claim that statins can induce autophagy and suppress cancer, and that autophagy may be involved in cancer metastasis; however, more direct evidence is needed to support the argument that statin-induced autophagy is responsible for the statin-induced decrease in cancer cell metastasis. Therefore, future research should evaluate the correlation between statin-induced autophagy and statin-induced antimetastatic effects. Although some explanations have been proposed, it is still difficult to determine when autophagy prevents cancer metastasis and when it promotes it.

Similarly, previous studies have shown that statins modulate the immune system by interfering with dendritic cell (DC) maturation and function [213,214,215]. Statins inhibit MHC-II expression, which is induced by cytokines and antigen-presenting-cell (APC) costimulatory molecules, and prevent antigen presentation to CD4 T cells. In addition, statins inhibit Th1 differentiation by inhibiting STAT-4 and increase the secretion of the anti-inflammatory cytokine Th2 by activating STAT-6 and GATA-3. Thus, statins suppress the secretion of proinflammatory cytokines and alter the profiles of T cells by changing the Th1/Th2 balance [216].

These immune-modulating effects of statins may be detrimental to maintaining effective, long-term antitumor immunity. In addition, questions remain as to whether statins can induce autophagy in immune cells and whether autophagy in immune cells can increase cancer progression. Furthermore, is it feasible for the activation of autophagy by statins to occur both in cancer cells and in the cells of the tumor microenvironment? Finally, the answers to these questions could help to clarify the promising potential of statins for reducing carcinogenic processes through autophagy. However, there is a need to perform further clinical studies to clarify and improve prevention practices or statin-based cancer treatment in the future.

## Figures and Tables

**Table 1:** In vitro and in vivo studies on the anticancer potential of atorvastatin via autophagy.

Statin	Cancer Type	In Vitro	In Vivo	Dosage	Observation
Atorvastatin	Breast cancer	MDA-MB-231 Cells	-	0,5, 1, 2, 4, 8 µM	Reduced the viability of cancer cells by inducing autophagy [25].
Atorvastatin	Breast cancer	MCF-7	-	5, 10, 20, 40 y 80 μM	Decreased the proliferation of breast cancer cells through the induction of both apoptosis and autophagy [184].
Atorvastatin	Ovarian cancer	Hey and SKOV3 cells	-	1–250 μM	Inhibited the growth of ovarian cancer cell lines associated with the induction of apoptosis, autophagy, cellular stress, and G1 cell-cycle arrest [19].
Atorvastatin	Cervical Cancer	SiHa and Caski Cells	Female BALB/c nude mice	0, 5, 10, y 20, 40, 80 μM (in vitro) 50 mg/kg (in vivo)	Reduced the viability of cervical cancer cells in vitro and in vivo by inducing apoptosis. ATO induced autophagy, and its inhibition was shown to enhance the anti-cancer effects of ATO on cervical cancer cells [24].
Atorvastatin	Digestive malignancies	HCC cells (Hep3B, HepG2 and Huh7) CRC cells (HCT116 wt, HCT116 p21)	Female BALB nude mice	50 μM (in vitro) 50 mg/kg (in vivo)	Inhibited cancer cell growth in vivo and in vitro by inducing apoptosis. ATO induced autophagy, and the pharmacological inhibition of autophagy was shown to enhance the anticancer effects of ATO in gastrointestinal malignancies [182].
Atorvastatin	Bladder Cancer	T24 and J28 Cells	-	0, 10, 20, 30, 40 y 50 μM	Enhanced ATP-induced apoptotic cell death in human bladder cancer cells in vitro through the pharmacological inhibition of autophagy [186].

**Table 2:** In vitro and in vivo studies on the anticancer potential of fluvastatin via autophagy.

Statin	Cancer Type	In Vitro	In Vivo	Dosage	Observation
Fluvastatin	Breast cancer	MCF-7	-	10 μM	Reduced cell viability through the depletion of lysosomal activities coupled with the accumulation of autophagosomes, leading to impaired autophagosome–lysosomal fusion in treated cells [187].
Fluvastatin	Lung adenocarcinoma	A549 and SPC-A-1 cells	Female nude mice BALB/c	10 μM (in vitro) 50 mg/kg (in vivo)	Suppressed bone metastasis from lung adenocarcinoma in vivo and in vitro by triggering autophagy through the p53–AMPK-mTOR pathway [175].
Fluvastatin	Lymphoma	A20 and EL4 cells	-	0–10 μM	Induced apoptosis in lymphoma cells by activating autophagy through increased LC3-II [188].

**Table 3:** In vitro and in vivo studies on the anticancer potential of lovastatin via autophagy.

Statin	Cancer Type	In Vitro	In Vivo	Dosage	Observation
Lovastatin	Malignant pleural mesothelioma	ACC-MESO-1 Cells	Mice NOD/SCID/γnull (NOG)	10 μM (in vitro) 12.5 mg/kg (in vivo)	Decreased viability and migration capacity of malignant pleural mesothelioma tumor cells by stimulating autophagy [189].
Lovastatin	Malignant peripheral nerve sheath tumor	NF90-8 and ST88-14 Cells	-	500 nM	Suppressed viability of cancer cells by inducing non-apoptotic cell death and altering autophagy flux [190].
Lovastatin	Human mesothelioma	Cancer cells ZL55	-	2, 8 µM	Reduced the viability of tumor cells by inducing autophagy [191].
Lovastatin	Primary effusion lymphoma (PEL)	BC3 and BCBL1 cells	-	3, 10, 30 µM	Reduced the survival of PEL cells by triggering apoptotic cell death through the inhibition of autophagic flux [192].

**Table 4:** In vitro and in vivo studies on the anticancer potential of pitavastatin via autophagy.

Statin	Cancer Type	In Vitro	In Vivo	Dosage	Observation
Pitavastatin	Pancreatic cancer	ASPC-1 and PANC-1 cells	-	10 µM	Decreased cell viability by triggering apoptosis, necrosis, and autophagy [193].
Pitavastatin	Melanoma	Human melanoma cells A375 and WM115	-	0–5 µM	Induced autophagy and decreased viability of cancer cells [194].

**Table 5:** In vitro and in vivo studies on the anticancer potential of rosuvastatin via autophagy.

Statin	Cancer type	In Vitro	In Vivo	Dosage	Observation
Rosuvastatin	Papillary thyroid carcinoma	B-CPAP and Nthy-ori 3-1 cells	-	12,5, 18,5, 25, 50, 100 y 200 µM	Decreased the proliferation and induction of cell death in thyroid cells in a dose- and time-dependent manner [195].

**Table 6:** In vitro and in vivo studies on the anticancer potential of simvastatin via autophagy.

Statin	Cancer type	In Vitro	In Vivo	Dosage	Observation
Simvastatin	Breast cancer	MDA-MB-231 cells	-	0.50 µM	Reduced the viability of breast cancer cells by inhibiting autophagy [196].
Simvastatin	Glioma	U251 and C6 cells	-	0–50 µM	Increased the antiglioma effect through the inhibition of the AMPK-dependent autophagic response [197].
Simvastatin	Brain cancer	GBM cells	-	0–20 µM	Inhibited temozolomide-induced autophagy flux by blocking autophagolysosome formation [198].

## Data Availability

Data sharing is not applicable.
